# Trade-Offs between Growth Rate, Tree Size and Lifespan of Mountain Pine (*Pinus montana*) in the Swiss National Park

**DOI:** 10.1371/journal.pone.0150402

**Published:** 2016-03-01

**Authors:** Christof Bigler

**Affiliations:** Forest Ecology, Institute of Terrestrial Ecosystems, Department of Environmental Systems Science, ETH Zurich, Zurich, Switzerland; Chinese Academy of Forestry, CHINA

## Abstract

A within-species trade-off between growth rates and lifespan has been observed across different taxa of trees, however, there is some uncertainty whether this trade-off also applies to shade-intolerant tree species. The main objective of this study was to investigate the relationships between radial growth, tree size and lifespan of shade-intolerant mountain pines. For 200 dead standing mountain pines (*Pinus montana*) located along gradients of aspect, slope steepness and elevation in the Swiss National Park, radial annual growth rates and lifespan were reconstructed. While early growth (i.e. mean tree-ring width over the first 50 years) correlated positively with diameter at the time of tree death, a negative correlation resulted with lifespan, i.e. rapidly growing mountain pines face a trade-off between reaching a large diameter at the cost of early tree death. Slowly growing mountain pines may reach a large diameter and a long lifespan, but risk to die young at a small size. Early growth was not correlated with temperature or precipitation over the growing period. Variability in lifespan was further contingent on aspect, slope steepness and elevation. The shade-intolerant mountain pines follow diverging growth trajectories that are imposed by extrinsic environmental influences. The resulting trade-offs between growth rate, tree size and lifespan advance our understanding of tree population dynamics, which may ultimately improve projections of forest dynamics under changing environmental conditions.

## Introduction

Across different taxa of trees, a within-species trade-off between growth rates and lifespan has been observed with slow-growing trees reaching a longer lifespan than fast-growing trees. For intermediate shade-tolerant to shade-tolerant tree species, several studies suggest a negative association between radial growth rates and maximum ages derived from living trees [1–3] or between growth rates (e.g. early growth during the first decades of a tree’s life) and lifespans derived from dead trees [4–6]. For shade-intolerant species, radial growth data from living trees indicate that old trees tend to grow slower than young trees [3,7,8]; however, the generality of a trade-off between radial growth and lifespan has recently been questioned [9]. The relationship between growth rates and tree lifespans may constitute an important component of tree population dynamics, which eventually entails consequences for the aboveground forest biomass and productivity [10,11] as well as for the terrestrial carbon cycle [12].

Differing growth rates of trees and the resulting variability in tree size are mainly the outcome of environmental influences such as (i) light intensities that are affected by stand structure, (ii) site conditions that are determined by topography, nutrient or soil water availability, and (iii) climate variability [13–15]. In general, growth rates increase along gradients of increasing resources (i.e. nutrients, water and radiation) [16]. These environmentally induced phenotypic responses result in diverging growth trajectories that entail benefits and costs for the trees [17–19]. Trees with rapid growth generally benefit from early maturation [20], early canopy attainment [21], increased competitiveness [13], fast escape from herbivory and fire at ground level [22], and high survival [23]. Adverse effects of rapid tree growth include lower annual reproduction [24], reduced structural and chemical defenses [25], low wood density that facilitates stem breakage and pathogen attacks [26], lower sectoriality and thus fewer functionally independent transport paths [27], and higher maintenance costs [22]. Rapidly growing, tall trees may further suffer from increased hydraulic resistance [28]. The previously mentioned shorter lifespan as a response to rapid early growth may be considered as delayed costs, resulting in a shorter reproduction time. Long-lived trees benefit from longer reproduction time and probably higher lifetime reproductive output [20,29]. Furthermore, reproduction is distributed over a longer time frame, which allows part of the offspring to survive during favorable periods and compensate for high mortality losses during adverse periods [22]. However, due to external mortality factors such as disturbances, climatic extreme events, infestation by fungi or sustained strong shading from neighboring trees, trees are at a high risk of dying before the onset of reproduction.

The overall aim of this study was to investigate the relationships between radial growth, tree size and lifespan of mountain pines (*Pinus montana* Miller) in a subalpine forest landscape. Mountain pine is a shade-intolerant tree species, which resists frost and low temperatures, and grows on calcareous, acid, moist and dry sites. I hypothesize that growth rates of mountain pine are negatively associated with lifespan, but positively related to tree size. Tree size and lifespan are expected to be positively associated. Since a few studies have identified differential effects of topography on lifespan [1,5,6], the combined effects of aspect, elevation and slope steepness were quantified. To verify whether the relationship between growth rates and lifespan was affected by changes in climate conditions, the association between temperature and precipitation during the growing period and early growth was tested. At high-elevation sites, growth rates are expected to be limited by temperature, while precipitation is expected to be of minor importance.

## Materials and Methods

### Study area

The study was conducted in the Swiss National Park (SNP), which is located in south-eastern Switzerland. The SNP was founded in 1914 as a strict nature reserve and extends over 170.3 km^2^. The high elevations, which range from 1400 to 3173 m a.s.l., and the continental climate result in a mean annual air temperature of 0.7°C (10.7°C in July), a precipitation total of 793 mm (107 mm in July), 73.5 cm of snowfall in December and 180 snow cover days (measured from 1981 to 2010 at the climate station Buffalora; 1968 m a.s.l., 46°39’N, 10°16’E). The rendzina soils have formed on dolomite and limestone, which account for 80% of the SNP. The area is covered by 28% forests, which have been shaped by century-long land and forest use [30]. Seventy-three percent of the forests consist of mountain pine (*Pinus montana*) and dwarf mountain pine (*Pinus mugo*), 11% of mixed Swiss stone pine (*Pinus cembra*)–European larch (*Larix decidua*). The tree line is located at ca. 2250 m a.s.l. Due to the harsh climate and the typically shallow soils, tree growth tends to be slow resulting in relatively small-sized trees.

### Selection of study plots

Twenty study plots were selected within a 28 km^2^ area with the perimeter bounded by 46°41’N, 10°10’E, 46°39’N and 10°16’E [31]. The selection of the plots was conducted using the ArcGIS software (ESRI, Redlands, USA), a digital elevation model and colour infrared aerial photographs. Forest stand polygons had been previously delineated based on the aerial photographs [32] and were homogeneous regarding tree species composition and standing dead wood. Five plots were selected from each north-, south-, east- and west-facing sites in forest stand polygons that included ≥ 60% mountain pine and ≥ 1–5% standing dead wood. The centers of the polygons were located between 1900 and 2100 m a.s.l., the mean slopes of the polygons ranged between 20° and 40°.

### Field sampling

Two hundred dead standing mountain pines were sampled in the field. At each of the 20 study plots, 10 trees with ≥ 10 cm DBH (diameter at breast height) were sampled within ca. 30 × 30 m [31]. Trees were classified as dead if the crown was lacking green needles. The field sampling was aimed at selecting background mortality [33], thus only trees were considered that had not been killed by disturbances such as rock fall, avalanches, mudflow, fire, storm or snow breakage. From each dead tree, two increment cores were extracted with an increment borer along the contour line in opposite directions. The cores were taken at a height of 84.0 ± 23.8 cm (mean ± standard deviation), which avoids reaction wood and biased radial growth rates that are more likely to occur at the stem base. From eight out of 200 trees only one core could be extracted because of advanced wood decay. In the field, further measurements were taken from these 200 dead mountain pines including DBH (measured at 130 cm), tree height, elevation, slope steepness and aspect (north-based azimuth). At 19 of 20 study plots, increment cores from two living mountain pines were taken, which were used to derive a tree-ring chronology.

### Laboratory methods

The increment cores were glued on core mounts and sanded on a belt sander with increasing grit from 180 to 600. The tree-ring widths of the increment cores were measured at a resolution of 0.01 mm using a LINTAB 5 measurement bench and the TSAP-Win software (both from Rinntech, Heidelberg, Germany). The tree rings were crossdated visually and quantitatively with the COFECHA software [34]. The underlying principle of crossdating is related to the common year-to-year growth pattern across trees from the same region, which is induced by the climate variability [35]. The tree rings of the dead mountain pines were crossdated with a tree-ring chronology that was developed from 32 living mountain pines (6 of the 38 living trees were omitted because of missing rings) and 12 dead mountain pines [31]. If a core missed the pith, the distance and number of missed rings between pith and the first complete ring on the core were estimated [36]. The cambial age of each core was estimated as the sum over the number of tree rings and missed rings. For the following analyses, only crossdated cores without eroded outermost rings were considered that missed the pith by less than 20 mm and by less than 20 rings.

### Measures of lifespan, early growth and tree size

The lifespan of a tree was approximated by the cambial age of the core with the higher cambial age if two cores were available; otherwise, the cambial age from the only core was used. Early growth was estimated as the mean ring width over the first 50 years (including the distance and number of missed rings between pith and first ring) from the core with the higher cambial age if two cores were available; otherwise, early growth of the only core was used. Restricting the period to the first 50 years for calculating early growth reduced the effect of the intrinsic age trend in the tree-ring series and allowed to include the youngest trees in the sample. For each core, the DBH inside bark was calculated as DBH_ib_ = 2 × $\sum_{\text{i}=1}^{\text{n}}{\text{rw}}_{\text{i}}$, where rw_i_ is the ring width of the i-th tree ring. Because the diameter was measured in the field with bark where present and because of eccentric growth of the tree stem, the DBH was in many cases larger than the DBH_ib_. Tree size was represented by the DBH_ib_.

### Data analysis

To test whether early growth was influenced by climate variability, which in turn might have an effect on lifespan, early growth was correlated with mean temperatures and precipitation sums, respectively, from May to September calculated over the same 50-year periods. Monthly temperature data from 1780 to 2008 and monthly precipitation data from 1801 to 2003 were available from the 5 × 5 minutes interpolated HISTALP (Historical Instrumental Climatological Surface Time Series of the Greater Alpine Region) climate data [37,38] and were extracted for a grid point (46°40’N, 10°10’E, 1823 m a.s.l.) nearby the study plots.

The combined effects of early growth and topography on lifespan of the mountain pines were modelled using log-linear mixed-effects models [39]:

$$\text{log}(y_{tp})=\alpha +\sum \beta_{k}x_{k,tp}+a_{p}+\varepsilon_{tp}$$ (1)

$$a_{p}∼Normal(0,\sigma_{p}^{2})$$

and

$$\varepsilon_{tp}∼Normal(0,\sigma^{2})$$

where log(y_tp_) is the log-transformed lifespan of tree t in plot p, α the intercept, β_k_ the fixed-effects parameter of variable x_k_ and *x*_*k*,*tp*_ the value of tree t in plot p for variable x_k_. The linear combination ∑*β*_*k*_*x*_*k*,*tp*_ represents any subset of all possible combinations of predictor variables (see below). The random effect a_p_ is normally distributed with variance $\sigma_{p}^{2}$ and accounts for the between-plot variability of the intercept; ε_tp_ are normally distributed residual errors with variance σ^2^. The models based on this structure converged and diagnostic plots did not show deviations from model assumptions, unlike other model structures such as generalized linear mixed-effects models with Poisson distribution.

An information-theoretic model selection approach was applied [40]. Models with the predictor variables “early growth” (unit: mm yr^-1^), “EW” (east-west gradient), “NS” (north-south gradient), “elevation” (elevation; unit: m a.s.l.) and “slope” (slope steepness; unit: °) were fitted. The east-west gradient was derived as EW = sin(aspect/360×2π) with east-facing sites assuming a value of 1 and west-facing sites -1. The north-south gradient was derived as NS = cos(aspect/360×2π) with north-facing sites assuming a value of 1 and south-facing sites -1. All possible combinations of zero up to five variables resulted in 32 (2^5^) models, which were compared using the AICc (Akaike Information Criterion correcting for small sample sizes; [39]):

$$AIC_{c}=-2\times \text{log}(likelihood)+2k\times \frac{n}{n-k-1}$$ (2)

where k is the number of parameters and n the number of observations. The models were ranked by increasing order of AIC_c_, i.e. the model with the lowest AIC_c_ was deemed the best-fitting model across the 32 models. For each model, the Akaike weight was calculated, which is the relative likelihood of the model given the data [40]:

$$w_{i}=\frac{\text{exp}\left(-\frac{1}{2}\Delta AIC_{c}^{i}\right)}{\sum_{j=1}^{32}\text{exp}\left(-\frac{1}{2}\Delta AIC_{c}^{j}\right)}$$ (3)

where $\Delta AIC_{c}^{i}$ is the AIC_c_ difference between model i and the model with the lowest AIC_c_. The Akaike weight w_i_ may be interpreted as the probability that model i is the best-fitting model within the selection of 32 models.

Because different combinations of fixed effects but with the same random effect (Eq 1) were compared, the models were fitted using maximum likelihood (ML; [40,41]). Since ML tends to underestimate variance components ($\sigma_{p}^{2}$ and *σ*^2^ in Eq 1; [39]), the best-fitting model was finally estimated using restricted maximum likelihood (REML; [40,41]).

The data analysis was conducted using the R software (version 3.0.2; [42]). Model fitting and selection were based on the R packages “lme4”, “lmerTest” and “MuMIn”; the figures were generated using the “ggplot2” package.

### Ethics statement and data availability

This research was conducted in the SNP, a strictly protected wilderness. Permission for field sampling was obtained from the Park management and the Research Committee of the SNP. The tree-ring chronology is publicly available from the International Tree-Ring Data Bank (dataset “SWIT283”, Swiss National Park, Grisons, Switzerland; http://www.ncdc.noaa.gov/paleo/study/16844). Data related to the individual trees are deposited in the public Dryad Digital Repository (doi:10.5061/dryad.d2680). Interpolated HISTALP climate data are available from the Department of Climate Research within the Central Institute for Meteorology and Geodynamics in Vienna, Austria (http://www.zamg.ac.at/histalp).

## Results

**Fig 1:**
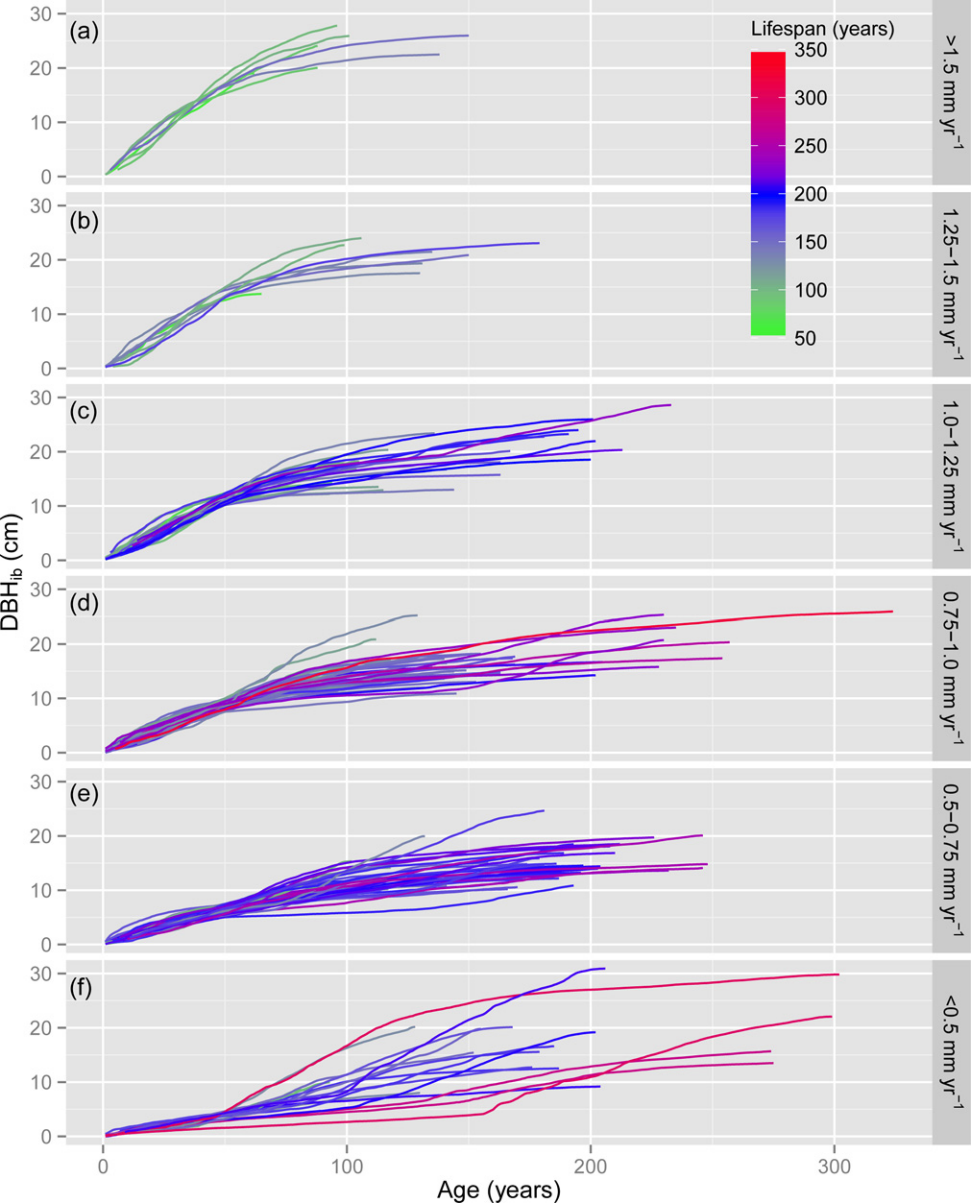
Development of tree size with age. Tree size is represented by DBH_ib_ (diameter at breast height inside bark); the color gradient indicates the lifespan of the mountain pines. Trees were assigned to classes of early growth (mean ring width over the first 50 years): (a) > 1.5 mm yr^-1^ (n = 7 trees); (b) 1.25–1.5 mm yr^-1^ (n = 8 trees); (c) 1.0–1.25 mm yr^-1^ (n = 27 trees); (d) 0.75–1.0 mm yr^-1^ (n = 54 trees); (e) 0.5–0.75 mm yr^-1^ (n = 46 trees); (f) <0.5 mm yr^-1^ (n = 18 trees).

The dead mountain pines in the SNP with a tree size of ≥ 10 cm DBH reached lifespans of 55 to 324 years, with 19% of the sampled tree population getting older than 200 years (Fig 1). Almost 22% of the trees measured > 20 cm DBH_ib_ and ca. 6% measured > 25 cm DBH_ib_ (maximum 30.9 cm). While mountain pines with the highest early growth (> 1.5 mm yr^-1^) tended to show continuously decreasing growth rates with increasing age (Fig 1A), many trees with the lowest early growth (< 0.5 mm yr^-1^) experienced abrupt growth releases following a 50- to 150-year period of slow growth (Fig 1F). In general, growth rates over the first few decades of a tree’s life were a strong determinant of tree size (DBH_ib_) at the time of tree death and of lifespan (Fig 1).

**Fig 2:**
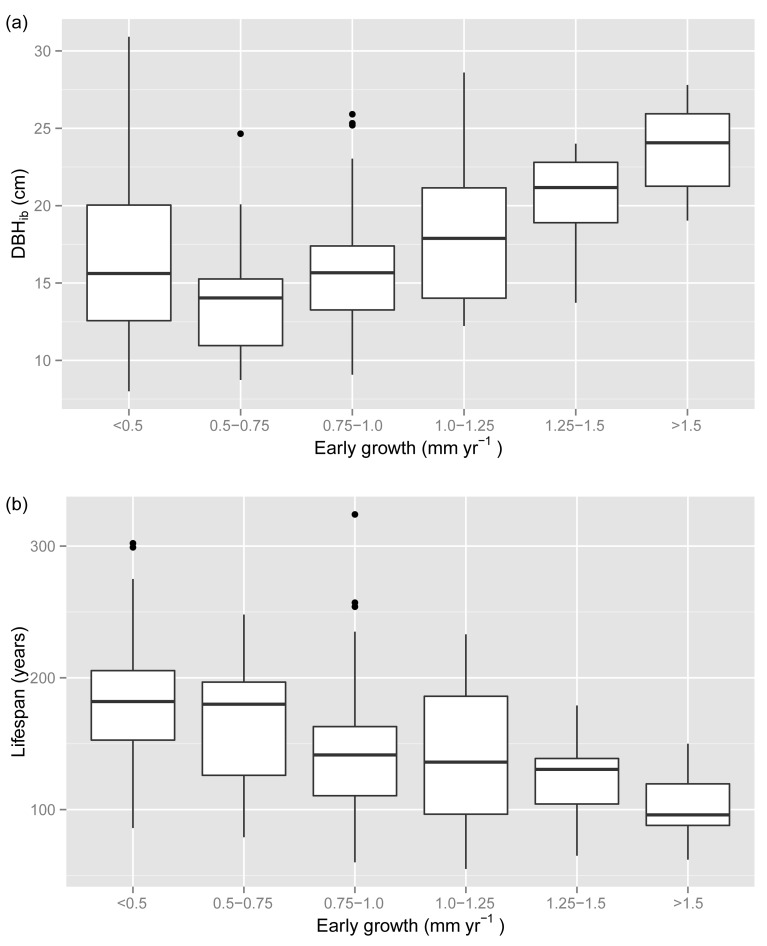
Distribution of tree size and lifespan versus early growth. Boxplots of (a) DBH_ib_ (diameter at breast height inside bark) at the time of tree death; and (b) lifespan. The variables are plotted for different classes of early growth (mean ring width over the first 50 years; n = 160 trees).

Tree size (DBH_ib_) correlated positively with early growth (Spearman’s rank correlation ρ = 0.39, P < 10^−6^, n = 160; S1 Fig). However, the largest trees (DBH_ib_ > 20 cm) showed no significant correlation between DBH_ib_ and early growth (ρ = 0.15, P = 0.39, n = 35). The DBH_ib_ declined from 23.6 ± 3.3 cm (mean ± standard deviation) for trees with > 1.5 mm yr^-1^ early growth to 14.0 ± 3.9 cm for trees with 0.5–0.75 mm yr^-1^ early growth, but increased to 16.7 ± 6.6 cm for trees with < 0.5 mm yr^-1^ (Fig 2A).

The lifespan of the mountain pines correlated negatively with early growth (ρ = -0.36, P < 10^−5^, n = 160; S1 Fig) and decreased from 188.9 ± 64.4 years for trees with < 0.5 mm yr^-1^ early growth to 103.3 ± 30.6 years for trees with > 1.5 mm yr^-1^ early growth (Fig 2B). Lifespan showed a positive correlation with tree size (DBH_ib_) based on all tree sizes (ρ = 0.34, P < 10^−4^, n = 160; S1 Fig). However, the largest trees (DBH_ib_ > 20 cm) showed no significant correlation between lifespan and DBH_ib_ (ρ = 0.11, P = 0.54, n = 35).

**Fig 3:**
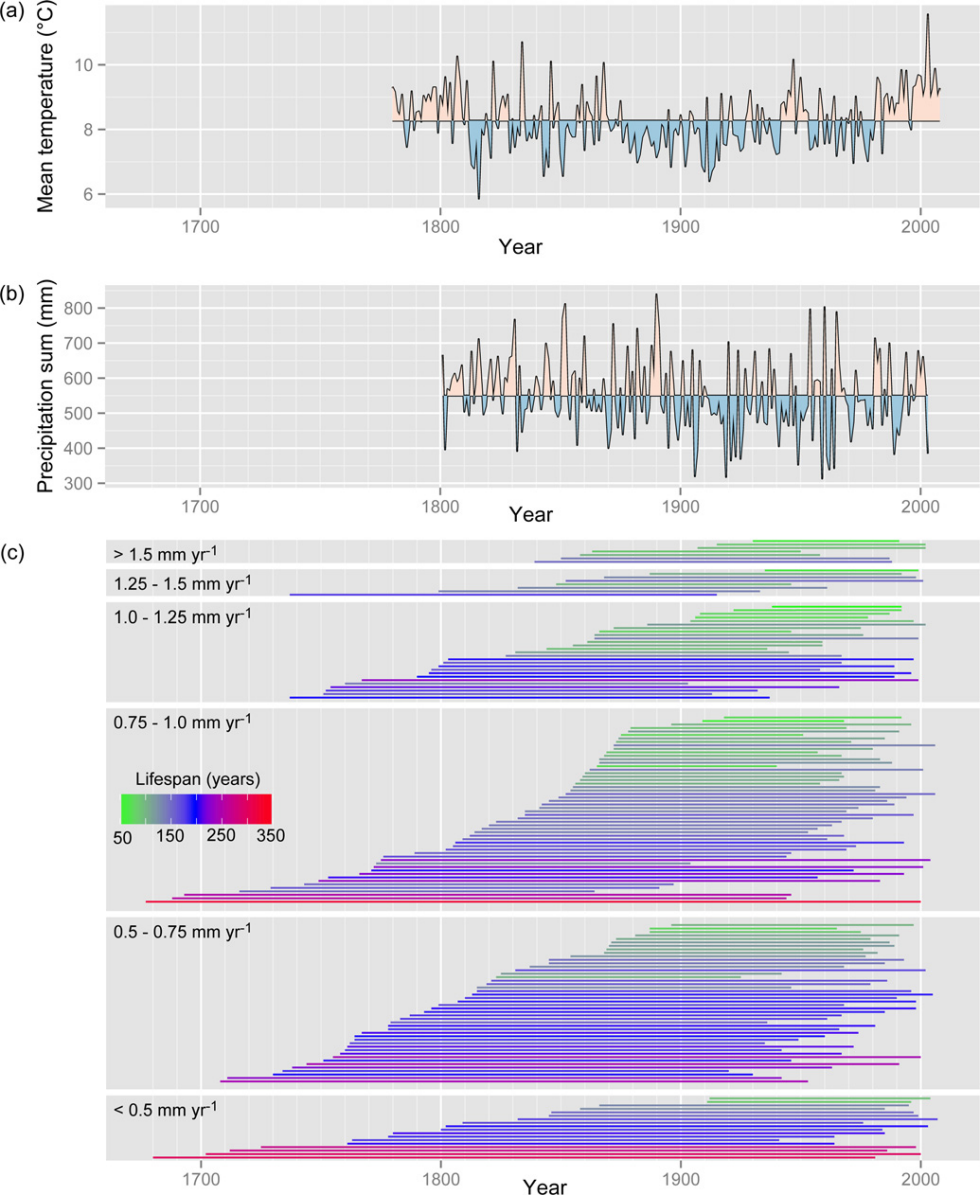
Comparison of temperature and precipitation with establishment and mortality of mountain pines. (a) Mean temperatures (May to September) from 1780 to 2008. Shaded colors indicate values above and below the overall mean temperature (8.27°C); (b) precipitation sums (May to September) from 1801 to 2003. Shaded colors indicate values above and below the overall mean precipitation (550 mm); (c) line plot of crossdated increment cores. Each horizontal line extends from the year of establishment (represented by the formation year of the first tree ring, corrected for missed rings) to the approximate year of mortality (represented by the formation year of the last tree ring) of a dead mountain pine. The same number of trees and classes of early growth as in Fig 1 are shown. Within each class of early growth, the trees were ordered according to the year of establishment. The color gradient indicates the lifespan of the trees.

The sampled mountain pines established between 1677 and 1938 (Fig 3C). Short-lived mountain pines established in more recent times than long-lived mountain pines (e.g. trees with a lifespan < 100 years established after 1844; Fig 3C). Early growth of trees that established since 1780 (i.e. since the beginning of the temperature series) was not correlated with the mean temperature from May to September (Fig 3A) during the corresponding 50-year periods (ρ = -0.042, P = 0.65; n = 114). Trees that established since 1801 (i.e. since the beginning of the precipitation series) did not show a significant correlation between early growth and precipitation from May to September (Fig 3B) during the corresponding 50-year periods (ρ = -0.178, P = 0.08; n = 101). The within-plot variability of early growth (i.e. growth rates during establishment) was relatively large for many plots (S2 Fig, S1 Table). Mortality dates, which were approximated by the year of formation of the outermost tree ring, occurred between 1864 and 2007 (Fig 3C).

The effects of early growth and topography on lifespan of mountain pines were quantified using a model selection procedure based on the AIC_c_ (Eq 2), which yielded for nine out of 32 models an Akaike weight (Eq 3) ≥ 0.1% (Table 1). Because the mixed-effects model with the lowest AIC_c_ (model 32) accounted for almost 60% of the Akaike weights and thus outperformed the remaining models, the following explanations refer to this model only. Accordingly, lifespan of mountain pines decreases with increasing early growth and along a gradient from west- to east-facing sites (Table 2; S1 and S3 Figs). Lifespan increases along a gradient from south- to north-facing sites and with increasing elevation and slope steepness (Table 2, S1 Fig). The comparison between observed and predicted lifespans using a leave-one-out model validation resulted in a positive Pearson’s correlation (r = 0.66, P < 2.2×10^−16^). Though observed lifespans > ca. 220 years tended to be underestimated, the diagnostic plots of the residuals and random effects did not indicate any violations of model assumptions. The variance inflation factors (VIFs < 1.4 for any predictor variable) and the Spearman’s rank correlations between predictor variables (-0.16 ≤ ρ ≤ 0.46; S1 Fig) indicate absence of multicollinearity [41].

**Table 1:** Comparison of linear mixed-effects models for predicting lifespan of mountain pines.

Model	Variables	AIC_c_	ΔAIC_c_	Akaike weight (%)
Model 32	Early growth + EW + NS + elevation + slope	16.8	0.00	59.9
Model 16	Early growth + EW + NS + slope	19.1	2.31	18.9
Model 24	Early growth + EW + NS + elevation	20.2	3.41	10.9
Model 8	Early growth + EW + NS	22.2	5.44	3.9
Model 28	Early growth + EW + elevation + slope	22.7	5.89	3.1
Model 20	Early growth + EW + elevation	23.0	6.25	2.6
Model 4	Early growth + EW	28.0	11.23	0.2
Model 12	Early growth + EW + slope	28.4	11.57	0.2
Model 30	EW + NS + elevation + slope	30.5	13.72	0.1

Shown are the predictor variables of the mixed-effects models (Eq 1), the AIC_c_ (Eq 2), the ΔAIC_c_ (AIC_c_ difference to model with lowest AIC_c_) and the Akaike weight (Eq 3). The models were fitted to data from 160 trees in 20 plots using maximum likelihood (ML). Only models with an Akaike weight ≥ 0.1% are shown (9 of 32 models). Abbreviations of variables: Early growth = mean ring width over the first 50 years (mm yr^-1^); EW = east-west gradient; NS = north-south gradient; elevation = elevation (m a.s.l.); slope = slope steepness (°).

**Table 2:** Description of model 32 (see Table 1).

Variable	Fixed effects			Random effects
	Estimate	SE	P	SD
Intercept	1.3736	1.7681	0.4490	0.1114
Early growth	-0.2692	0.0681	0.0001	
EW	-0.2263	0.0444	0.0001	
NS	0.1645	0.0505	0.0045	
Elevation	0.0018	0.0009	0.0559	
Slope	0.0079	0.0035	0.0321	
Residual error				0.2314

For the fixed effects, estimates (α and β_k_ in Eq 1), standard errors (SE) and P values (P) are shown. For the random effects, standard deviations (SD) are shown (σ_p_ for the intercept and σ for the residual error in Eq 1). The log-linear mixed-effects model (Eq 1) was fitted using restricted maximum likelihood (REML). For a description of the variables see Table 1.

## Discussion

### Development of tree size in response to growth rates

The mountain pines in the SNP follow a range of distinct growth trajectories (Fig 1). While < 10% of the trees experienced high early growth (> 1.25 mm yr^-1^), 50% show intermediate growth (0.75–1.25 mm yr^-1^) and 40% slow growth (< 0.75 mm yr^-1^). These varying growth rates likely resulted from differences in resource availability contingent on both site conditions and forest stand structure [15,43–45]. The relatively large within-plot variability in early growth (S2 Fig, S1 Table) suggests that stand structure varied even on a small spatial scale (e.g. most trees in plot “SNP.East.22” established between 1855 and 1868, but showed strongly differing growth rates) and changed during time (e.g. the decreasing early growth along time in “SNP.West.39” may indicate an increase in stand closure), respectively. The negative effect of intra-specific competition on growth rates of mountain pines has been demonstrated for two stands in the SNP along up to 70 years of stand development [46]. Particularly forest gap dynamics resulting from wind throw or root rot such as *Armillaria* sp. or *Heterobasidion annosum* [47,48], but also extensive logging before the foundation of the SNP [30] created openings large enough to allow for high early growth [49]. Related to the limited potential of crown expansion from neighboring conifers, many of these rapidly growing mountain pines reached a DBH_ib_ > 20 cm (Figs 1 and 2A), i.e. high early growth rates are generally a safe strategy to quickly reach a competitive tree size and attain the forest canopy [21,50]. Smaller forest gaps created by snow breakage, individual tree mortality or felling of single trees allowed mountain pines to grow initially at intermediate growth rates [51]. These gaps tend to fill in more rapidly than larger gaps, i.e. growth rates slow down due to increasing tree competition [46], which results in smaller sized trees compared to trees with higher growth rates (Figs 1 and 2A). Mountain pines with slow early growth were likely growing initially in relatively dense stands [46,51], however, more than 25% reached a DBH_ib_ > 20 cm as a result of strong release effects (Fig 1F). Abrupt growth releases are generally caused by background or disturbance-induced mortality, or by the felling of neighboring trees (e.g. established for mountain pines in the SNP; [49]), which results in a rapid increase of resources [52]. Thus large tree size may be attained not only by rapidly growing trees, but also by initially slowly growing trees through growth releases and extended lifespan [18].

### Trade-off between early growth and lifespan

The lifespan of mountain pines that reached ≥ 10 cm DBH is negatively associated with early growth (Fig 2B, S3 Fig, Table 2), i.e. trees with high early growth die relatively young, while slowly growing trees tend to reach a longer lifespan. Within-species trade-offs between growth rates and lifespan or tree age, respectively, have been revealed for shade-intolerant to shade-tolerant conifer and deciduous species across North America and Europe (e.g. [1,3,6,7,53]). However, deviating findings were found for the shade-intolerant *Populus tremuloides* in a recent study from northern Arizona, which reports higher growth rates of surviving trees compared to dead trees [9]. Part of the disagreement may be related to some methodological issues (e.g. the dead trees were biased towards smaller and younger trees) and to the strong impact of severe drought and heat resulting in rapid dieback [54], i.e. strong extrinsic mortality factors may override intrinsic trade-offs [55].

### Potential biases induced by sampling, environmental changes and logging

The observed trade-off between early growth and lifespan may have been affected by several potential biases induced by (1) the field sampling, (2) directed environmental changes and (3) past logging activities. The following two issues are related to the sampling of the trees. First, short-lived trees tend to have established in more recent times than long-lived trees. For instance, mountain pines in the SNP that survived for < 100 years established since the mid-19th century whereas mountain pines > 250 years old established before 1726 (Fig 3C). This apparent decrease in lifespan with later establishment partly reflects the fact that short-lived mountain pines, which established before the mid-19th century, had been likely decayed or removed during logging activities [56]. Second, sampling trees larger than a fixed size threshold (e.g. ≥ 10 cm DBH as in this study) results in including rapidly growing trees that tend to have established in more recent times than trees with slow early growth (Fig 3C; see [50,57]). Decreasing the DBH threshold would on the one hand mainly result in sampling more short-lived trees along the entire range of early growth rates (Fig 1, S3 Fig), which would decrease the effect size of early growth on lifespan (Table 2). On the other hand, some rapidly growing short-lived trees or some long-lived trees with sustained slow growth might be included in the sample as well, which would increase the effect size of early growth.

The unequal distribution of rapid-growing mountain pines that established in more recent times and slow-growing mountain pines that established in earlier times (Fig 3C) may have been caused as well by changing environmental conditions related to climate change, increasing atmospheric CO_2_ concentrations and increasing nitrogen deposition [10,58–60]. First, increased growth rates seem not to originate from changes in temperature or precipitation as indicated by the non-significant correlations between early growth and mean temperature or precipitation during the growing period, respectively (Fig 3). In fact, the first 50 years of quite a few trees with high early growth overlapped with the distinct cool period from 1870 to 1941, whereas the first 50 years of several trees with slow early growth overlapped with the warm period from 1780 to 1811 (Fig 3). This somewhat unexpected result with respect to the lack of a temperature effect may be explained by superimposing effects of other temporally varying influences such as stand structure (S2 Fig). Second, until the mid-19th century, the atmospheric CO_2_ concentration has been relatively stable reaching 285 ppm (parts per million) in 1850, followed by a rise to 296 ppm in 1900, 311 ppm in 1950 and 370 ppm in 2000 ([61]; recent data retrieved from the National Oceanographic and Atmospheric Administration, NOAA, at http://www.esrl.noaa.gov/gmd/ccgg/trends/). However, even a strong CO_2_ increase has no detectable effect on ring widths of mountain pine as shown in a 9-year free-air experiment (CO_2_ enrichment from 380 ppm to 575 ppm) at the tree line in Davos, Switzerland [62]. Third, global atmospheric deposition of reactive nitrogen has increased from 1860 to 1900 by ca. 25%, by more than 100% until 1950 and by more than 400% until 2000 [63]. Nitrogen deposition in the SNP has reached 4.8 kg ha^-1^ yr^-1^ (mean from 1999 to 2007) [64], which is expected to result only in a weak growth stimulation [65]. Since many trees with high early growth established before 1900 (Fig 3C), i.e. when only weak increases in CO_2_ and nitrogen deposition were reached, the apparent increase in early growth is unlikely to be caused by increased atmospheric CO_2_ concentration or nitrogen fertilization.

Logging activities before the foundation of the SNP in 1914 may have resulted in selective felling of trees with suitable wood qualities such as large size or straight stems. Historical documents that reach back to the 14th century suggest that most trees in the SNP have been cut for charcoal burning (e.g. used in ore mining), for export to the saline in Hall (Austria), for lime kiln and as firewood in local households [30]. Thus, it cannot be ruled out that these logging activities affected the size and age structure of the stands, notably by removing the biggest and oldest trees [1]. Assuming that if old trees with high early growth existed (i.e. trees that would be observed in the top right corner in S3 Fig), there would have been a fair chance to sample some of these trees. However, six of 15 trees with high early growth formed the outermost tree ring between 1915 and 1961 and reached lifespans of 88 to 179 years (Fig 3C), which indicates that these trees potentially had the chance to extend their lifespans by another ca. 50 to 90 years.

### Effects of topography on lifespan

The lifespan of mountain pines is further contingent on site conditions represented by topographical gradients (Table 2, S1 Fig). The lower lifespan on south-facing sites may be explained by higher radiation and resulting soil desiccation, which agrees with decreased lifespans of *Picea engelmannii* and *Abies lasiocarpa* on south-facing slopes in subalpine forests of Colorado [6]. The slight increase in lifespan with increasing elevation also confirms elevational effects on lifespan of upper storey *Picea abies* in the northern Swiss Alps [5] and on maximum ages of *Fagus sylvatica* in the eastern Alps and central Apennines [1]. Some of the most rapidly growing and short-lived mountain pines occur on flatter slopes (S1 Fig). While increased growth rates on flatter slopes may be explained through higher resource availability (e.g. reduced run-off of water, deeper soils and higher availability of soil moisture and nutrients), which in turn affects lifespan, there still remains a significant effect of slope steepness after eliminating the effect of early growth (Table 2). The increase in lifespan from east- to west-facing slopes is not readily explained with differences in radiation or soil desiccation. Part of the topographical effects may be related to previous logging activities [30], e.g. sites at higher elevation or on steeper slopes as well as certain aspects may have experienced less frequent or less intense cuttings.

### Potential explanations of the trade-off between growth and lifespan

The trade-off between early growth and lifespan will be discussed in the context of (1) resource allocation patterns and (2) size-related physiological changes. In general, plant growth rates and net assimilation rates (i.e. balance between carbon gain from photosynthesis and carbon loss from respiration) are assumed to follow a sigmoid curve along gradients of resource availability, while secondary metabolites related to defense or stress tolerance are assumed to follow a parabolic function (S4 Fig). These resource allocation patterns have been reflected in the growth-differentiation balance (GDB) hypothesis [66,67]. Applying the GDB hypothesis to the mountain pines in the SNP would imply that in environments with high to very high resources (e.g. in large gaps or in moist and nutrient-rich sites) growth receives a high allocation priority, because it is a highly resource-demanding process [68]. Consequently, trees with rapid growth but reduced secondary metabolism are assumed to prevail (S4 Fig), which benefit from enhanced resource acquisition and increased competitiveness at the cost of reduced defense and stress tolerance and thus increased mortality from herbivores, pathogens or environmental stress. At low to intermediate levels of resource availability (e.g. in relatively dense stands or in nutrient-deficient sites with low soil moisture), growth rates are constrained to a higher degree than net assimilation rates, thus surplus assimilates are allocated to secondary metabolism [68]. Consequently, trees with relatively low growth but high secondary metabolism are expected to dominate (S4 Fig), which show enhanced resistance to herbivores [69] and pathogens [70] as well as increased stress tolerance. At very low to low levels of resource availability (e.g. under very dense canopies), net assimilation and growth are highly constrained (S4 Fig), thus these trees will be outcompeted by neighboring trees and are prone to attacks by herbivores and pathogens [66]. Evidence of a strongly increased tree mortality risk associated with very low growth rates has been provided by a large number of studies (e.g. [71–73]).

For an improved explanation of the trade-off between growth and lifespan, the GDB hypothesis may be complemented with size-related changes in tree physiology [28,74]. Increased tree size has been associated with reduced growth rates, increased mortality and reduced fertility [75,76]. These size-related changes are assumed to be induced by increased hydraulic resistance in taller trees [28], reduced nutrient availability in the soil due to the long-term sequestration into biomass [74] and increased respiratory load due to the higher amount of respiring tissue compared to the photosynthesizing tissue [77]. These mechanisms may provide a complementary explanation, why mountain pines with the most rapid early growth die young when reaching a large diameter (Fig 1A), while some of the initially most slowly growing mountain pines die old after reaching a large diameter (Fig 1F). However, relatively many slowly growing trees die before reaching a large diameter (Fig 1E and 1F), i.e. size-related changes account only for some of the variability in lifespan.

## Conclusions

Trees follow diverging growth trajectories that are largely imposed by extrinsic environmental influences. The site- and stand-specific resource availability requires trees to adjust their allocation strategies, which results in vast differences in growth rates and tree sizes. As exemplified by the shade-intolerant mountain pines in the SNP, lifespan and tree diameter at the time of tree death are already partially determined during an early stage. Rapidly growing mountain pines follow a relatively narrow band of growth trajectories and likely reach a large diameter at the cost of reduced lifespan. In contrast, slowly growing mountain pines follow diverging growth trajectories with some trees reaching a large diameter and long lifespan, while other trees die early at a small tree size. Part of these latter differences may be explained by the competitiveness and life expectancy of neighboring trees. The observed distinct effects of tree growth and topography on tree lifespan should not conceal the fact that most trees die before reproduction starts or before reaching a certain size, often due to the impact of extrinsic mortality factors such as competition, frost, soil desiccation, herbivores or pathogens. Overall, the resulting trade-offs between growth rate, tree size and lifespan advance our understanding of tree population dynamics, which may ultimately improve projections of forest dynamics under changing environmental conditions.

## Supporting Information

S1 Fig Relationships between lifespan, early growth, DBH_ib_ and topographical variables. Shown are scatter plots (panels below diagonal) and Spearman’s rank correlations (panels above diagonal) between lifespan, early growth (mean ring width over the first 50 years), DBH_ib_ (diameter at breast height inside bark), EW (east-west gradient with 1 indicating east-facing sites and -1 indicating west-facing sites), NS (north-south gradient with 1 indicating north-facing sites and -1 indicating south-facing sites), elevation and slope steepness (n = 160 trees). The distributions of the variables are shown in the diagonal. The red lines are smoothers derived from locally weighted polynomial regressions.
(PDF)

S2 Fig Plot-specific variability of early growth. For each of the 20 study plots, a scatter plot between early growth (mean ring width over the first 50 years) and the first year of the corresponding 50-year period is shown (n = 160 trees). The rows correspond to the four plot aspects (from top to bottom: east, north, south, west). Each panel is labelled with a plot identifier (e.g. “SNP.East.22”; see S1 Table).
(PDF)

S3 Fig Relationship between early growth, lifespan and tree size. Shown is a scatter plot between early growth (mean ring width over the first 50 years) and lifespan (shown on log-transformed scale). Dots (n = 160 trees) are scaled with tree size (DBH_ib_, diameter at breast height inside bark). The fitted blue regression line (prediction) is based on model 32 (Table 2) with the remaining variables fixed at their mean values. The 95% CI (confidence interval) is based on parametric bootstrapping with 1000 repetitions.
(PDF)

S4 Fig Hypothetical change in net assimilation, relative growth rate and secondary metabolites along a gradient of resource availability. The figure is adapted from [66] and reflects the growth-differentiation balance (GDB) hypothesis. The curves for net assimilation, relative growth rate and secondary metabolites are not based on measurements, but just serve to visualize the suggested change in resource allocation with increasing resources. The resource gradient was arbitrarily divided into sectors of different resource availabilities.
(PDF)

S1 Table Summary table of study plots. The variable “plot” lists the plot identifiers; “number of trees” describes the number of trees used in the analysis (total and separately for the six classes of early growth). The variables “aspect”, “slope” (slope steepness), “elevation”, “lifespan”, “DBH_ib_” (diameter at breast height inside bark), and “early growth” (mean ring width over the first 50 years) are based on the trees used in the analysis (shown are mean ± standard deviation, SD). Mean and SD for the variable “aspect” were calculated using circular statistics in the R package “circular”.
(PDF)
