# Supplementary figures and images for: Trade-Offs between Growth Rate, Tree Size and Lifespan of Mountain Pine (Pinus montana) in the Swiss National Park
Source: PLoS One. 2016 Mar 1;11(3):e0150402. doi: 10.1371/journal.pone.0150402 (PMC4773076; doi:10.1371/journal.pone.0150402)

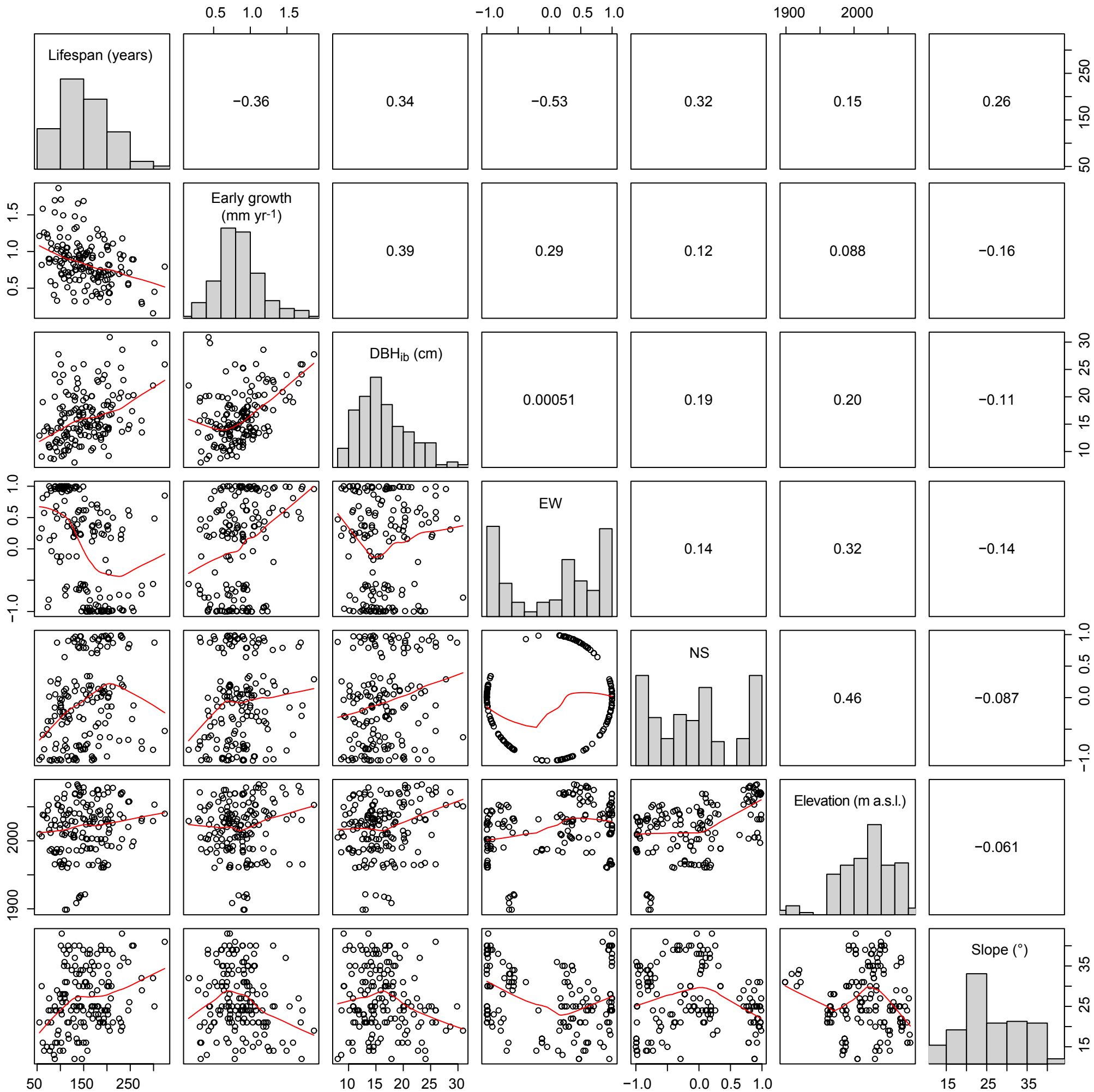

Supplement: S1 Fig — Shown are scatter plots (panels below diagonal) and Spearman’s rank correlations (panels above diagonal) between lifespan, early growth (mean ring width over the first 50 years), DBHib (diameter at breast height inside bark), EW (east-west gradient with 1 indicating east-facing sites and -1 indicating west-facing sites), NS (north-south gradient with 1 indicating north-facing sites and -1 indicating south-facing sites), elevation and slope steepness (n = 160 trees). The distributions of the variables are shown in the diagonal. The red lines are smoothers derived from locally weighted polynomial regressions. (PDF) [file pone.0150402.s001.pdf]

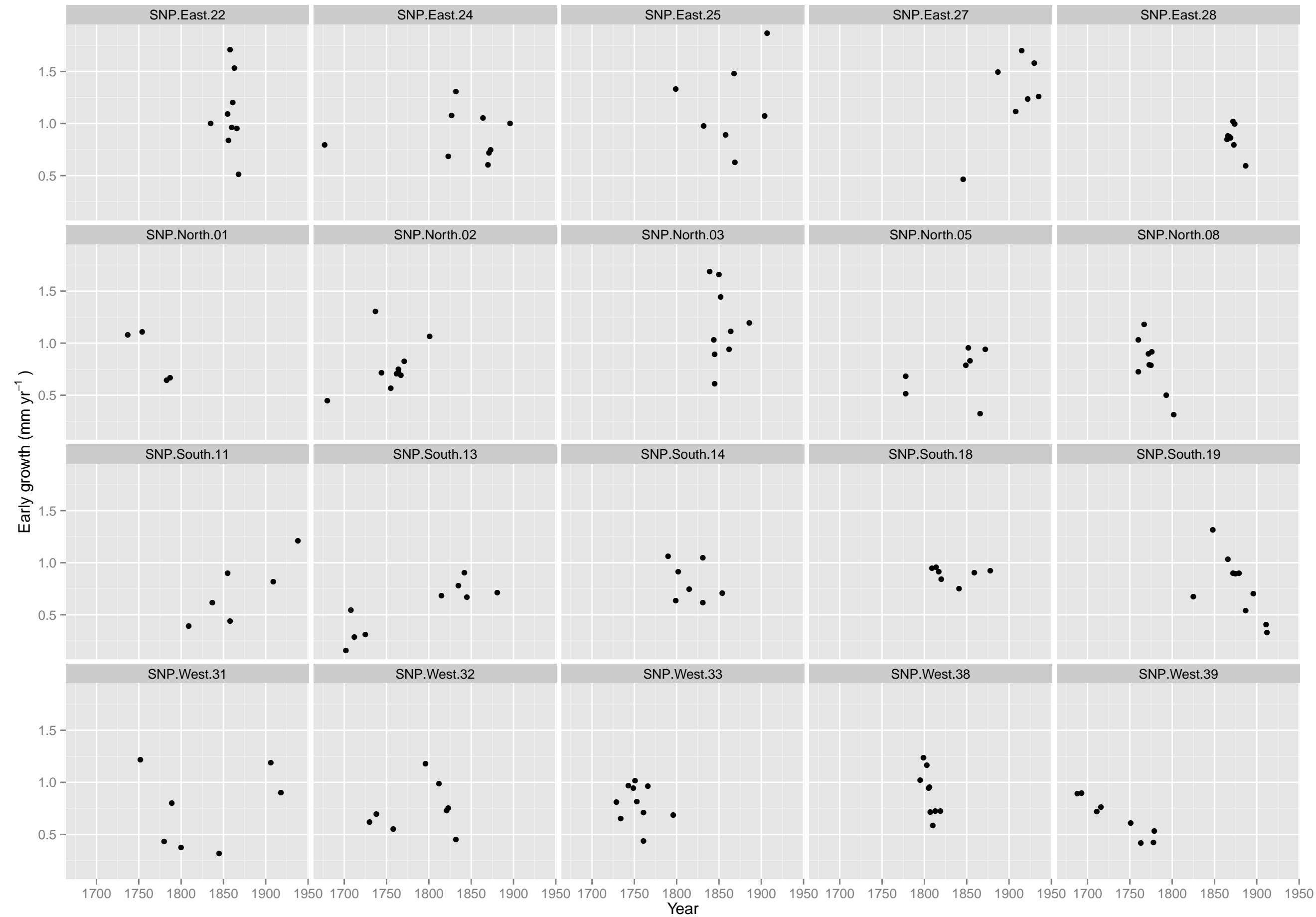

Supplement: S2 Fig — For each of the 20 study plots, a scatter plot between early growth (mean ring width over the first 50 years) and the first year of the corresponding 50-year period is shown (n = 160 trees). The rows correspond to the four plot aspects (from top to bottom: east, north, south, west). Each panel is labelled with a plot identifier (e.g. “SNP.East.22”; see S1 Table). (PDF) [file pone.0150402.s002.pdf]

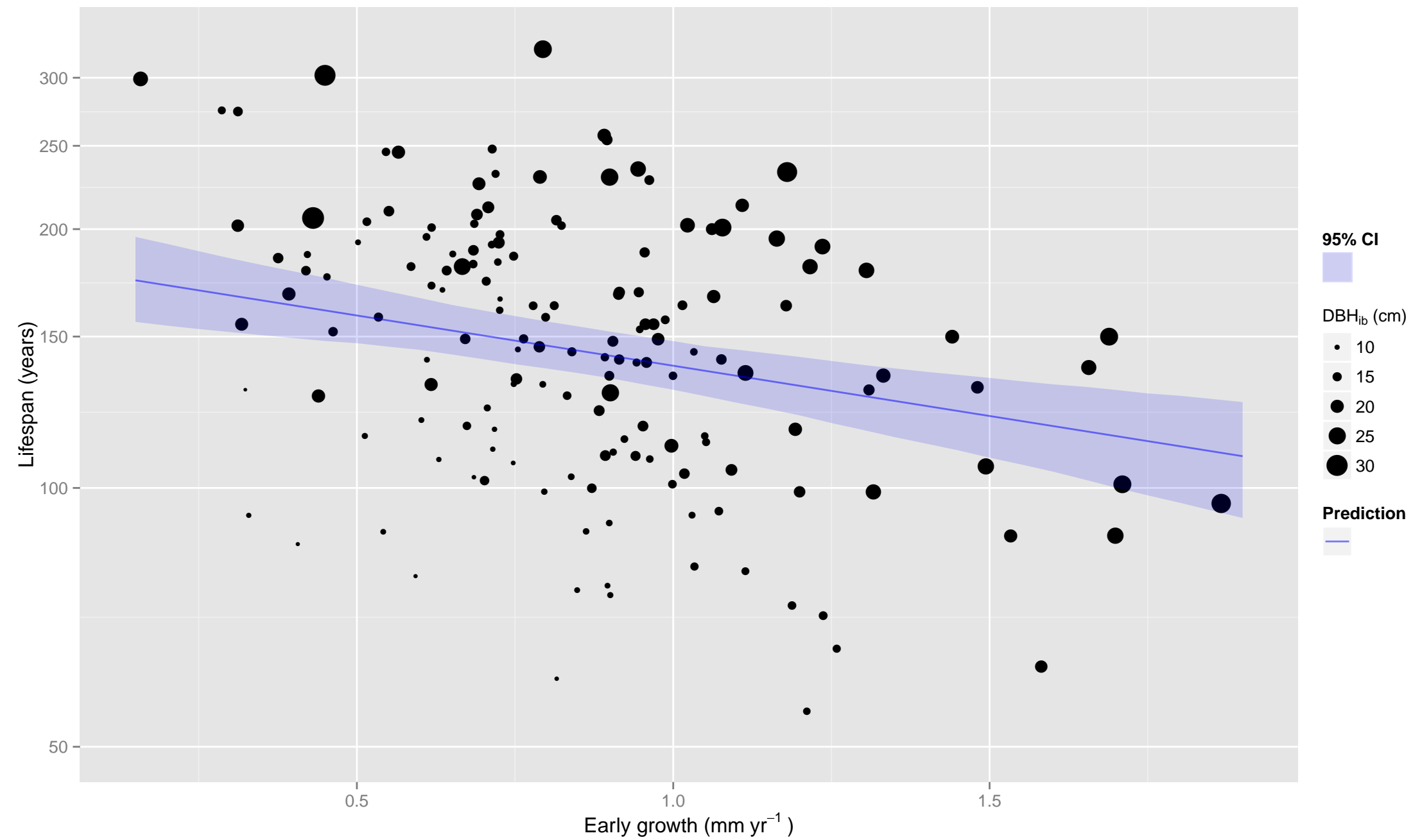

Supplement: S3 Fig — Shown is a scatter plot between early growth (mean ring width over the first 50 years) and lifespan (shown on log-transformed scale). Dots (n = 160 trees) are scaled with tree size (DBHib, diameter at breast height inside bark). The fitted blue regression line (prediction) is based on model 32 (Table 2) with the remaining variables fixed at their mean values. The 95% CI (confidence interval) is based on parametric bootstrapping with 1000 repetitions. (PDF) [file pone.0150402.s003.pdf]

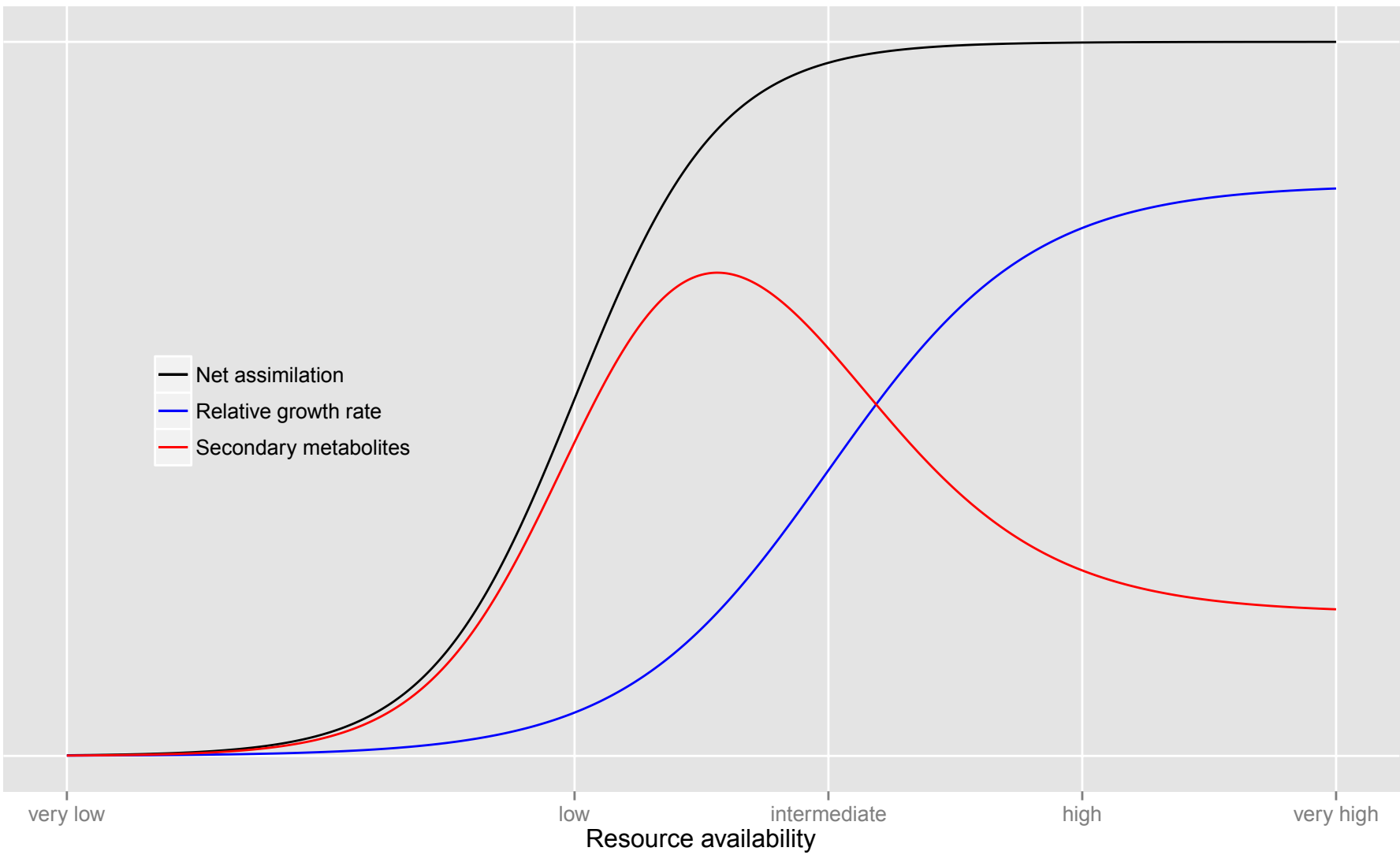

Supplement: S4 Fig — The figure is adapted from [66] and reflects the growth-differentiation balance (GDB) hypothesis. The curves for net assimilation, relative growth rate and secondary metabolites are not based on measurements, but just serve to visualize the suggested change in resource allocation with increasing resources. The resource gradient was arbitrarily divided into sectors of different resource availabilities. (PDF) [file pone.0150402.s004.pdf]
