# Supplementary material for: Trade-Offs between Growth Rate, Tree Size and Lifespan of Mountain Pine (Pinus montana) in the Swiss National Park
Source: PLoS One. 2016 Mar 1;11(3):e0150402. doi: 10.1371/journal.pone.0150402 (PMC4773076; doi:10.1371/journal.pone.0150402)
Supplement: S1 Table — The variable “plot” lists the plot identifiers; “number of trees” describes the number of trees used in the analysis (total and separately for the six classes of early growth). The variables “aspect”, “slope” (slope steepness), “elevation”, “lifespan”, “DBHib” (diameter at breast height inside bark), and “early growth” (mean ring width over the first 50 years) are based on the trees used in the analysis (shown are mean ± standard deviation, SD). Mean and SD for the variable “aspect” were calculated using circular statistics in the R package “circular”. (PDF) [file pone.0150402.s005.pdf]

Plot	Number of trees							Aspect (°)	Slope (°)	Elevation (m)	Lifespan (years)	DBH_{ib} (cm)	Early growth (mm yr^{-1})
	Total	<0.5 mm	0.5-0.75 mm	0.75-1.0 mm	1.0-1.25 mm	1.25-1.5 mm	>1.5 mm						
SNP.East.22	9	0	1	4	2	0	2	104.1 ± 0.1	24.7 ± 2.8	1965.6 ± 3.4	108.0 ± 13.4	16.6 ± 4.6	1.09 ± 0.36
SNP.East.24	9	0	4	2	2	1	0	99.0 ± 0.2	40.0 ± 1.4	2041.8 ± 4.0	139.6 ± 70.3	14.2 ± 5.4	0.89 ± 0.23
SNP.East.25	7	0	1	2	1	2	1	81.0 ± 0.1	27.7 ± 8.2	2059.8 ± 7.1	117.4 ± 21.0	18.5 ± 5.6	1.18 ± 0.41
SNP.East.27	7	1	0	0	2	2	2	126.2 ± 0.2	16.0 ± 3.3	2032.3 ± 4.9	89.1 ± 31.5	17.7 ± 4.7	1.26 ± 0.41
SNP.East.28	8	0	1	6	1	0	0	91.0 ± 0.2	25.3 ± 1.4	2015.5 ± 5.6	97.8 ± 16.0	14.1 ± 4.1	0.86 ± 0.13
SNP.North.01	4	0	2	0	2	0	0	34.0 ± 0.1	24.8 ± 3.3	2076.4 ± 2.0	193.5 ± 16.4	21.7 ± 4.6	0.87 ± 0.25
SNP.North.02	10	1	6	1	1	1	0	26.0 ± 0.3	24.7 ± 1.6	2045.6 ± 12.2	210.9 ± 42.3	18.5 ± 5.0	0.78 ± 0.24
SNP.North.03	9	0	1	2	3	1	2	30.1 ± 0.1	21.4 ± 5.2	2074.5 ± 7.6	134.1 ± 18.2	18.2 ± 5.5	1.17 ± 0.36
SNP.North.05	7	1	2	4	0	0	0	22.7 ± 0.4	23.7 ± 8.6	2005.1 ± 4.6	151.6 ± 34.2	15.1 ± 3.5	0.72 ± 0.23
SNP.North.08	9	1	2	4	2	0	0	15.9 ± 0.1	20.7 ± 1.0	2069.7 ± 1.5	190.7 ± 37.4	17.8 ± 6.2	0.79 ± 0.26
SNP.South.11	6	2	1	2	1	0	0	165.2 ± 0.1	26.7 ± 3.7	2008.7 ± 3.7	112.0 ± 44.8	17.9 ± 5.8	0.73 ± 0.31
SNP.South.13	9	3	4	2	0	0	0	222.0 ± 0.1	32.7 ± 3.4	2025.2 ± 6.8	205.2 ± 68.6	15.3 ± 3.2	0.56 ± 0.25
SNP.South.14.	7	0	4	1	2	0	0	163.9 ± 0.1	34.7 ± 1.0	2029.3 ± 4.1	154.4 ± 31.1	13.8 ± 3.0	0.82 ± 0.19
SNP.South.18	7	0	0	7	0	0	0	218.0 ± 0.0	31.9 ± 1.5	1911.5 ± 9.7	133.7 ± 15.9	15.0 ± 2.2	0.89 ± 0.07
SNP.South.19	10	2	3	3	1	1	0	174.6 ± 0.3	16.2 ± 3.0	1985.4 ± 2.1	97.1 ± 17.7	13.4 ± 4.1	0.77 ± 0.30
SNP.West.31	7	3	0	2	2	0	0	233.4 ± 0.2	19.3 ± 3.4	2048.2 ± 4.1	147.6 ± 53.1	18.6 ± 6.6	0.75 ± 0.38
SNP.West.32	8	1	4	2	1	0	0	265.2 ± 0.4	26.8 ± 3.6	1989.3 ± 2.4	180.5 ± 28.6	14.6 ± 3.4	0.75 ± 0.24
SNP.West.33	10	1	3	5	1	0	0	274.0 ± 0.2	36.0 ± 3.2	2030.5 ± 9.1	195.5 ± 27.8	15.8 ± 3.8	0.80 ± 0.18
SNP.West.38	9	0	4	2	3	0	0	275.2 ± 0.1	22.9 ± 1.4	1964.3 ± 3.2	185.8 ± 13.2	17.9 ± 4.3	0.90 ± 0.22
SNP.West.39	8	2	3	3	0	0	0	260.8 ± 0.2	39.5 ± 1.6	1999.5 ± 4.7	201.5 ± 41.7	15.4 ± 2.5	0.66 ± 0.19
